# Sanitary Blocks of Hospitals

**Published:** 1920-04-03

**Authors:** Keith D. Young

**Affiliations:** 17 Southampton Street, Bloomsbury, London, W.C. 1.


					SANITARY BLOCKS OF HOSPITALS.
To the Editor of Tiie Hospital.
' tI{> May 1 S?ly a few words in reply to your article
u"<ler the above head ?
? 1,1 the example described of a suite of sanitary rooms
Ringed exactly on the principle" 1 recommend, a
P?Ult is made of the fact that the draught under the
,,,rs in the direction of the ward was a very strong one.
? would a cross-ventilated lobby prevent this.' It
V ' ertainly no part of my plan that air should be drawn
*1 0 the ward after first passing over " a little collection
I sPecimens." In a properly contrived receptacle for
edpans waiting inspection, such a thing could not
aPpen. The American experience cannot be lightly dis-
missed with a reference to artificial ventilation. Modern
practice in America, without exception, is, against the
cut-off lobby, and the adoption of artificial ventilation
is by no means universal. I can see no reason applied
to the planning of sanitary offices in hospitals which
is not equally cogent, in relation to private houses.?
1 ours faithfully,
Keith D. Young.
17 Southampton Street, Bloomsbury, .London, W.C. 1.
March 26, 1920.
[The letter printed above refers to an article on page 615
in last week's issue. Mr. Keith Young's comment will
interest our readers, and we shall hope to refer to it
in a subsequent issue.?Ed. The Hospital.]

				

